# Typical phenotypes of patients with acute respiratory failure with and without COVID-19 and their relationship with outcomes: a cohort study

**DOI:** 10.5935/2965-2774.20230015-en

**Published:** 2023

**Authors:** Mirella Cristine de Oliveira, Rafaella Stradiotto Bernardelli, Amanda Christina Kozesinski-Nakatani, Joelle Turnes, Fernanda Baeumle Reese, Leandro Caramuru Pozzo, Rafael Alexandre de Oliveira Deucher, Caroline Uliana Rossi, Luana Alves Tannous, Álvaro Réa-Neto

**Affiliations:** 1 Centro de Estudos e de Pesquisas em Terapia Intensiva - Curitiba (PR), Brazil; 2 Hospital do Trabalhador - Curitiba (PR), Brazil; 3 Hospital das Nações - Curitiba (PR), Brazil; 4 Hospital Santa Casa de Curitiba - Curitiba (PR), Brazil; 5 Hospital INC - Instituto de Neurologia de Curitiba - Curitiba (PR), Brazil; 6 Hospital São Lucas - Curitiba (PR), Brazil

**Keywords:** COVID-19, Coronavirus infections, SARS-CoV-2, Epidemiology, Critical care, Intensive care units

## Abstract

**Objective:**

To compare, within a cohort of patients with acute respiratory failure, the phenotypes of patients with and without COVID-19 in the context of the pandemic and evaluate whether COVID-19 is an independent predictor of intensive care unit mortality.

**Methods:**

This historical cohort study evaluated 1001 acute respiratory failure patients with suspected COVID-19 admitted to the intensive care unit of 8 hospitals. Patients were classified as COVID-19 cases and non-COVID-19 cases according to real-time polymerase chain reaction results. Data on clinical and demographic characteristics were collected on intensive care unit admission, as well as daily clinical and laboratory data and intensive care unit outcomes.

**Results:**

Although the groups did not differ in terms of APACHE II or SOFA scores at admission, the COVID-19 group had more initial symptoms of fever, myalgia and diarrhea, had a longer duration of symptoms, and had a higher prevalence of obesity. They also had a lower PaO2/FiO2 ratio, lower platelet levels than non-COVID-19 patients, and more metabolic changes, such as higher levels of blood glucose, C-reactive protein, and lactic dehydrogenase. Patients with non-COVID-19 acute respiratory failure had a higher prevalence of chronic obstructive pulmonary disease/asthma and cardiopathy. Patients with COVID-19 stayed in the hospital longer and had more complications, such as acute kidney failure, severe acute respiratory distress syndrome and severe infection. The all-cause mortality rate was also higher in this group (43.7% in the COVID-19 group *versus* 27.4% in the non-COVID-19 group). The diagnosis of COVID-19 was a predictor of intensive care unit mortality (odds ratio, 2.77; 95%CI, 1.89 - 4.07; p < 0.001), regardless of age or Charlson Comorbidity Index score.

**Conclusion:**

In a prospective cohort of patients admitted with acute respiratory failure, patients with COVID-19 had a clearly different phenotype and a higher mortality than non-COVID-19 patients. This may help to outline more accurate screening and appropriate and timely treatment for these patients.

## INTRODUCTION

The coronavirus disease 2019 (COVID-19) pandemic imposed a burden on hospitals and was a leading cause of morbidity and mortality worldwide.^(1)^ Since COVID-19 is a new, aggressive disease and can be confused with other respiratory diseases, its specific clinical management is not well established, leading to longer hospitalizations, more frequent intensive care unit (ICU) stays, complications, and poor outcomes.^(2)^ While these aspects seem to have improved over the course of the pandemic,^(3,4)^ we still have a great deal to learn from what occurred and prepare for what may come.

In addition to substantial respiratory injury, i.e., acute respiratory failure, the virus can directly or indirectly promote extrapulmonary complications that affect almost all major systems (cardiovascular, gastrointestinal, renal, hepatic, endocrine, and nervous).^(5)^ Due to the seriousness of COVID-19, it has been estimated that approximately 32% of patients who are hospitalized for the disease may need intensive care.^(6)^ Furthermore, the mortality rate in intensive care is very high (19.6 - 40%).^(4,7)^ However, many other acute respiratory diseases have a similar clinical presentation and different therapeutic approaches. Therefore, it is essential to characterize these patients, identify possible risk factors, and develop strategies to improve their ICU care.

There is a shortage of comparative studies that focus on concomitant patients experiencing acute respiratory failure with and without COVID-19. Such studies usually rely on historical controls^(8-10)^ or small populations of patients solely on mechanical ventilation.^(11)^ It is important to identify the clinical differences between these two distinct populations upon admission to the hospital to make an early and accurate differential diagnosis. Additionally, knowledge of the evolving characteristics and potential complications over time of each group can provide insights into prognosis.

Therefore, the purpose of this study was to analyze the clinical data of 1,001 acute respiratory failure patients who were admitted to the ICU during the early stages of the pandemic in Brazil. This study aimed to compare the data of concomitant patients with and without COVID-19 and to identify any differences in their phenotypes. This study also aimed to determine whether COVID-19 was an independent predictor of ICU mortality.

## METHODS

This prospective cohort study included consecutive patients with acute respiratory failure secondary to suspected respiratory infection admitted to the ICU between March 11 and September 13, 2020, in 8 hospitals in Curitiba, Brazil. During this period, these hospitals had a maximum capacity of 225 beds exclusively for patients with acute respiratory failure and a strong suspicion of COVID-19. Of these, 124 were dedicated to public health care patients, 71 to private health care patients, and 30 to mixed health care patients.

This study was approved by the local ethics committee of the *Hospital INC -Instituto de Neurologia de Curitiba*, under protocol 2.899.18,8 on September 17, 2018. The same committee waived the requirement for informed consent, given the noninterventional design of this study and the fact that the data were collected from clinical records and without contact with the participants and the procedures performed in this study were part of routine care. All research procedures were conducted in accordance with the ethical standards of the institutional committee on human experimentation and with the Declaration of Helsinki of 1975 as revised in 2013. To ensure proper reporting, we utilized the Strengthening the Reporting of Observational Studies in Epidemiology (STROBE) guidelines for this study.

This study included patients over the age of 18 who were admitted to the ICU with acute respiratory failure caused by a suspected respiratory infection. These patients underwent a real-time polymerase chain reaction (RT-PCR) test to detect severe acute respiratory syndrome coronavirus 2 (SARS-CoV-2), which was collected through a nasopharyngeal swab. During the pandemic period, patients were screened for acute respiratory failure secondary to suspected respiratory infection using a set of clinical and radiological criteria that were regularly employed by the study institutions. If at least two of the clinical and radiological criteria were present, they were diagnosed with acute respiratory failure due to a secondary or suspected respiratory infection: at least one flu-like symptom, i.e., cough, runny nose, fever, or sore throat; at least two items from the modified quick-Sequential Organ Failure Assessment (qSOFA) scale (systolic blood pressure < 100mmHg, respiratory rate > 22bpm, decreased level of consciousness with Glasgow Coma Scale score < 15, and/or oxygen pulse saturation < 93%); and chest computed tomography (CT) scans with images suggestive of COVID-19 (ground-glass opacity and peripheral lesions distributed across both lungs) obtained in the first 48 hours after admission.^(12)^ Patients without complete daily follow-up records during their ICU stay were excluded from the cohort.

During the study period, some participating sites had to temporarily pause or end the inclusion of patients in the cohort due to a high number of admissions to the ICUs and overload of care. This decision was made to prioritize patient care and ensure the safety of the research team. Patients admitted to the ICUs when this study was on hold were not screened for this study. However, during the active periods of the sites, all patients were screened and included consecutively.

The patients were divided into a group with a diagnosis of COVID-19 confirmed by RT‒PCR (COVID-19 group) and another group in whom this diagnosis was refuted (non-COVID-19 group). To mitigate bias from false-negative test results, the non-COVID-19 group included only patients who had more than one negative RT‒PCR result or only one negative first RT‒PCR result if the patient had another diagnosis that was more likely than SARS-CoV-2 infection to explain the diagnosis of acute respiratory failure.

Data were obtained from electronic patient records and daily follow-up records of critically ill patients recorded at the bedside on paper and captured on an eCRF based on RedCap unique to this cohort. Demographic and clinical data were collected at ICU admission, and daily clinical and laboratory data and ICU outcomes were collected for all included patients. Clinical variables collected in the first 24 hours included comorbidities (also including the Charlson Comorbidity Index), symptoms and signs at admission, and duration of symptoms until ICU admission. The following variables were also collected from the medical records within the first 24 hours of hospitalization: mean arterial pressure, heart rate, respiratory rate, temperature, capillary glycemia, use of sedative drugs, level of respiratory support, use of vasoactive drugs, blood count laboratory test results, coagulation tests, renal and hepatic function, inflammatory markers, electrolytes, arterial blood gases and D-dimer levels. The Acute Physiology and Chronic Health Evaluation (APACHE II) classification system was used as a prognostic score based on data from the first 24 hours of hospitalization. Organ dysfunction attributed to the different systems was characterized by the SOFA score, with data collected daily until the outcome.

We also systematically analyzed treatments applied during hospitalization and complications such as pleural effusion, coagulation disorders (i.e., thromboplastin time [PT] with International Normalized Ratio [INR] > 1.5 and/or kaolin partial thromboplastin time (KPTT) > 45 seconds and/or platelets < 150,000 units/microliter), acute renal failure (assessed by the AKI-KDIGO), severe acute respiratory failure and secondary infections. Other important parameters evaluated were length of stay in the ICU, time and need for mechanical ventilation, level of advanced life support limitation at the time of outcome, and ICU mortality. Clinical status on the World Health Organization (WHO) 9-point ordinal scale was used in the first 24 hours and at the time of ICU outcome to classify patients in terms of respiratory compromise.

## Statistical analysis

Categorical variables are presented as absolute frequencies and percentages, quantitative variables with a normal distribution as means and standard deviations, and quantitative variables without a normal distribution as means, medians and interquartile ranges. Categorical variables were compared between the COVID-19 and non-COVID-19 groups using the chi-square test or Fisher’s exact test, as appropriate. Quantitative variables comparisons between groups were performed using Student’s t test for independent samples when data were normally distributed and the nonparametric Mann-Whitney U test when data were not normally distributed.

The odds ratio and respective 95% confidence interval (95%CI) of COVID-19 for mortality during the ICU stay were estimated by multivariate binary logistic regression models adjusted by age and Charlson Comorbidity Index score (representing comorbidities). We determined these confounding factors *a priori*, following recommendations for observational studies among critically ill patients.^(13)^ The Wald test was used to analyze the significance of each variable included in the models.

The level of statistical significance was set at 5%, and the data were analyzed using the statistical software IBM Statistical Package for the Social Sciences (SPSS), version 28.0 (SPSS Inc., Chicago, Illinois, USA). Imputation for missing data was not performed.

## RESULTS

**Figure 1:**
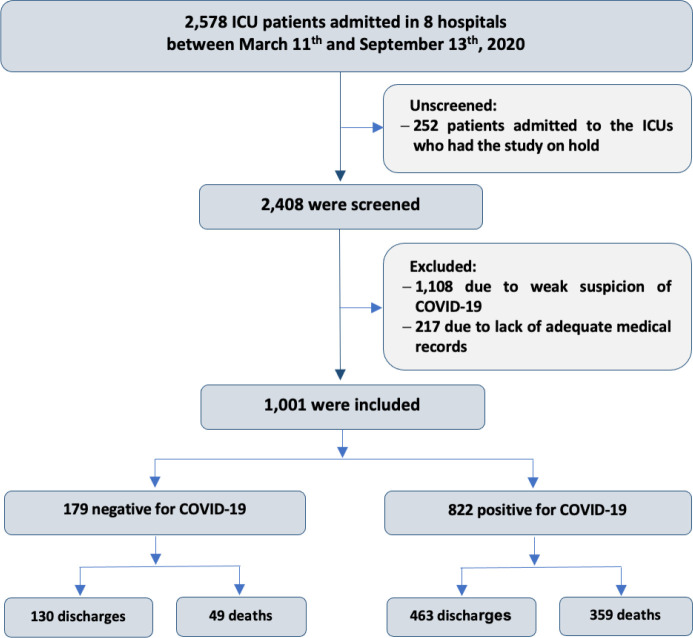
Flowchart of patients with severe acute respiratory syndrome with and without COVID-19 in terms of baseline characteristics and outcomes.

During the inclusion period, 2,578 patients were admitted to the ICUs of the eight hospitals. Of these patients, 1,001 were included because they met the three clinical-radiographic inclusion criteria, and all underwent PCR testing for SARS-CoV-2 on admission. After the results, the 822 individuals who were positive made up the COVID-19 group, and the 179 individuals who were negative made up the non-COVID-19 group. Figure 1 describes the flow of patients admitted to the ICUs until the final definition of the sample.

ICU - intensive care unit.

The non-COVID-19 group (n = 179) included the following diagnoses established to explain the diagnosis of acute respiratory failure: 41.3% had a clinical diagnosis of bacterial pneumonia; 22.4% cardiovascular diseases; 17.9% exacerbated chronic pneumopathies (asthma, chronic obstructive pulmonary disease [COPD], or pulmonary fibrosis); 5.0% sepsis of extrapulmonary etiology; 4.5% neurological diseases; 2.8% lung cancer; 2.2% pulmonary thromboembolism; 1.7% metabolic decompensation; 1.1% pneumonitis; and 1.1% tuberculosis.

At admission to the ICU, there were no significant differences between the COVID-19 and non-COVID-19 groups in terms of their APACHE II score, SOFA score, or baseline clinical status as classified by the 9-point ordinal scale. Additionally, the proportion of patients using invasive mechanical ventilation, vasoactive drugs and sedation at baseline was not significantly different between the two groups (Table 1).

**Table 1:** Comparison of baseline characteristics among patients admitted to the ICU with severe acute respiratory syndrome due to COVID-19 and other causes

Baseline characteristics	COVID-19 (n=822)	Non-COVID-19 (n=179)	p value
Time from symptom onset to ICU admission (days)^a^	7 (5 - 10)	4 (2 - 7)	< 0.001^*^
Age (years)	61 ± 15.8	64.4 ± 18.3	0.029†
Male sex	472 (57.4)	91 (50.8)	0.155‡
Body mass index^b^	29.3 ± 6.1	26.9 ± 5.6	< 0.001†
Comorbidities			
Cardiopathy	145 (17.6)	51 (28.5)	0.003‡
Systemic arterial hypertension	427 (51.9)	90 (50.3)	0.803‡
COPD/asthma	89 (10.8)	49 (27.4)	< 0.001‡
Chronic kidney disease	47 (5.7)	9 (5.0)	1‡
Diabetes	250 (30.4)	46 (25.7)	0.234‡
AIDS/HIV	10 (1.2)	2 (1.1)	1‡
Cancer	28 (3.4)	10 (5.6)	0.359‡
Obesity	246 (29.9)	29 (16.2)	0.001‡
Charlson Comorbidity Index	1 (0 - 2)	1 (0 - 2)	< 0.001^*^
Admission signs and symptoms			
Fever	420 (51.5)	64 (36.2)	0.002‡
Cough	498 (61.1)	108 (60.7)	0.497‡
Sore throat	77 (9.4)	19 (10.7)	0.573‡
Rhinorrhea	77 (9.4)	15 (8.4)	0.886‡
Sibilance	17 (2.1)	17 (9.6)	< 0.001‡
Chest pain	33 (4.0)	15 (8.4)	0.082‡
Myalgia/arthralgia	189 (23.2)	15 (8.4)	< 0.001‡
Fatigue	210 (25.8)	39 (21.9)	0.388‡
Dyspnea	706 (86.4)	154 (86.5)	0.905‡
Headache	81 (9.9)	11 (6.2)	0.152‡
Decreased level of consciousness	87 (10.7)	36 (20.3)	0.001‡
Abdominal pain	19 (2.3)	7 (3.9)	0.197‡
Vomit	91 (11.2)	15 (8.4)	0.418‡
Diarrhea	107 (13.1)	9 (5.1)	0.003‡
Clinical status on the 9-point ordinal scale at ICU admission			0.113§
3 - Hospitalized, no oxygen therapy	70 (8.5)	22 (12.3)	
4 - Hospitalized, oxygen by mask or nasal prongs	539 (65.6)	104 (58.1)	
5 - Hospitalized, noninvasive ventilation or high-flow oxygen	11 (1.3)	0 (0)	
6 - Hospitalized, intubated and on mechanical ventilation	91 (11.1)	23 (12.8)	
7 - Hospitalized, on mechanical ventilation and additional organ support (renal replacement therapy, vasoactive drugs or ECMO)	111 (13.5)	30 (16.8)	
Clinical and laboratory data from the 1^st^ 24 hours			
APACHE score	13 (8 - 19)	13 (9 - 19)	0.495^*^
SOFA score	3 (2 - 6)	4 (2 - 6)	0.362^*^
Higher mean arterial pressure^c^	101.5 ± 16.7	103.0 ± 20.1	0.367†
Lower mean arterial pressure^c^	75.8 ± 14.9	76.4 ± 16.3	0.597†
Heart rate^d^	89.3 ± 23.8	94.8 ± 25.4	0.005†
Respiratory frequency^e^	25.7 ± 7.7	24.2 ± 5.7	0.004†
Temperature^f^	36.7 ± 1.1	36.6 ± 0.8	0.336†
Sedation use	200 (24.3)	50 (27.9)	0,341‡
Glasgow Coma Scale score	15 (15 - 15)	15 (15 - 15)	< 0.001^*^
Vasoactive drug use	134 (16.3)	36 (20.1)	0.227‡
Invasive mechanical ventilation use	209 (25.4)	53 (29.6)	0.261‡
PaO_2_/FiO_2_	212.7 ± 132	264.1 ± 134.3	< 0.001†
PaO_2_/FiO_2_			< 0.001†
≥ 200	379 (46.1)	117 (65.4)	
Between 199 e 100	254 (30.9)	48 (26.8)	
< 100	189 (23.0)	14 (7.8)	
Hemoglobin (g/dL)^g^	12.9 ± 2.2	13.1 ± 2.3	0.155†
Hemoglobin < 10g/dL^g^	79 (9.6)	13 (7.3)	0.392‡
Leukocytes (cont./m^3^)^g^	9,971.0 ± 5,810.1)	12,308.9 ± 5,811.9)	< 0.001†
% Lymphocytes^h^	12 (7 - 18)	12 (8 - 18)	0.930^*^
% Neutrophils^i^	83 (76 - 88)	82 (77 - 88)	0.546^*^
% Hematocrit^j^	38.1 ± 5.8	39.1 ± 7.1	0.173†
% Hematocrit < 30%^g^	41 (9.6)	10 (8.7)	0.859‡
Platelets (cont./m^3^)^g^	215,436.2 (97,541.2)	208,571.1 (100,409.9)	0.397†
Platelets < 150,000 cont./m^3 g^	196 (23.8)	45 (25.3)	0.699‡
INR^k^	1.1 (1.0 - 1.2)	1.2 (1.1 - 1.3)	0.061^*^
KPTT (seconds)^l^	28.6 ± 6.9	28.2 ± 8.5	0.320†
Sodium (mEq/L)^m^	136.2 ± 6.9	136.7 ± 7.0	0.388†
Sodium < 130 mEq/L^m^	59 (7.7)	19 (10.9)	0.122‡
Potassium (mEq/L)^n^	4.2 ± 0.8	4.2 ± 0.8	0.841†
Potassium ≥ 5.5mEq/L^n^	43 (5.3)	11 (6.9)	0.466‡
Higher blood glucose (mg/dL)^o^	163 (124 - 230)	145 (116.5 - 184)	0.002^*^
Creatinine (mg/dL)^p^	0.90 (0.7 - 1.4)	1.0 (0.7 - 1.4)	0.342^*^
Creatinine > 1.2mg/dL^p^	259 (31.7)	59 (33.3)	0.658‡
Urea (ml/dL)^p^	43 (30.2 - 70)	48.5 (34 - 76)	0.093^*^
Total bilirubin (mg/dL)^q^	0.4 (0.3 - 0.6)	0.6 (0.4 - 0.9)	< 0.001^*^
Total bilirubin > 1.2mg/dL^q^	23 (3.6)	11 (9.5)	0.012‡
GOT (U/L)^r^	44 (31 - 67)	34 (21 - 65)	0.001^*^
GPT (U/L)^r^	35 (24 - 56)	24.5 (16 - 49)	< 0.001^*^
Lactate (mmol/L)^s^	1.5 (1.0 - 2.1)	1.5 (1.1 - 2.3)	0.456^*^
Lactate ≥ 2mmol/L^s^	236 (29.2)	55 (32.9)	0.354‡
C-reactive protein (mg/L)^t^	122.2 (69 - 183.7)	41.5 (15 - 122)	< 0.001^*^
LDH (U/L)^u^)	429.5 (343 - 592)	286 (203 - 430)	< 0.001^*^
D-dimer (ng/mL)^v^	1,081 (562 - 2,798)	1,602.6 (446 - 4,490.7)	0.785^*^
pH^x^	7,391 ± 0.114	7,382 ± 0.103	0.336†
pH < 7.33^x^	171 (21.8)	38 (22.9)	0.757‡
PaO_2_ (mmHg)	87.6 ± 32.9	95.7 ± 38.2	0.009†
PCO_2_ (mmHg)^w^	37.3 ± 10.0	41.2 ± 11.9	< 0.001†
HCO_3_ (mEq/L)^x^	22.4 ± 4.7	24.3 ± 5.9	< 0.001†
Base excess (mEq/L)^y^	-1.8 (-4.7 - 1.0)	-0.9 (-4.5 - 2.5)	0.007^*^
SaO_2_ (%)^z^	95 (92 - 97)	96 (94 - 98)	< 0.001^*^

ICU - intensive care unit; COPD - chronic obstructive pulmonary disease; ECMO - extracorporeal membrane oxygenation; APACHE II - Acute Physiology and Chronic Health Evaluation; SOFA - Sequential Organ Failure Assessment; PaO_2_ - partial pressure of oxygen; FiO_2_ - fraction of inspired oxygen; INR - International Normalized Ratio; KPTT - kaolin partial thromboplastin time; GOT - glutamic oxaloacetic transaminase; GPT - glutamic pyruvic transaminase; LDH - lactate dehydrogenase; PCO_2_ - partial pressure of carbon dioxide; HCO_3_ - bicarbonate; SaO_2_ - oxygen saturation.

Missing data:

a 92 in COVID-19 and 31 in non-COVID-19 group;

b 359 in COVID-19 and 75 in non-COVID-19 group;

c 7 in COVID-19 and 4 in non-COVID-19 group;

d 6 in COVID-19 and 3 in non-COVID-19 group;

e 38 in COVID-19 and 10 in non-COVID-19 group;

f 35 in COVID-19 and 14 in non-COVID-19 group;

g 1 in non-COVID-19 group;

h 407 in COVID-19 group and 19 in non-COVID-19 group;

i 417 in COVID-19 and 22 in non-COVID-19 group;

j 396 in COVID-19 and 64 in non-COVID-19 group;

k 532 in COVID-19 and 94 in non-COVID-19 group;

l 610 in COVID-19 and 113 in non-COVID-19 group;

m 12 in COVID-19 and 4 in non-COVID-19 group;

n 9 in COVID-19 and 4 in non-COVID-19 group;

o 73 in COVID-19 and 27 in non-COVID-19 group;

p 4 in COVID-19 and 2 in non-COVID-19 group;

q 183 in COVID-19 and 63 in non-COVID-19 group;

r 396 in COVID-19 and 80 in non-COVID-19 group;

s 15 in COVID-19 and 12 in non-COVID-19 group;

t 5 in COVID-19 and 5 in non-COVID-19 group;

u 516 in COVID-19 and 115 in non-COVID-19 group;

v 463 in COVID-19 and 99 in non-COVID-19 group;

x 36 in COVID-19 and 13 in non-COVID-19 group;

w 35 in COVID-19 and 13 in non-COVID-19 group;

y 320 in COVID-19 and 34 in non-COVID-19 group;

z 38 in COVID-19 and 13 in non-COVID-19 group.

* Nonparametric Mann-Whitney U test, p < 0.05 indicates statistical significance; † Student's t test for independent samples, p < 0.05 indicates statistical significance; ‡ Fisher's exact test, p < 0.05 indicates statistical significance; § chi-square test, p < 0.05 indicates statistical significance. Results are expressed as the medians (first quartile - third quartile), means ± standard deviations or n (%).

The COVID-19 group experienced a longer time between symptom onset and hospital admission (with a median of 7 days compared to 4 days for the non-COVID-19 group) and had a higher mean body mass index of 29.3 compared to 26.9 for the non-COVID-19 group. The COVID-19 group also reported a higher rate of obesity and symptoms such as fever, myalgia/arthralgia, and diarrhea than the non-COVID-19 group (Table 1).

Furthermore, the non-COVID-19 group had a higher mean age (64.4 *versus* 61) and a significantly greater proportion of patients with cardiomyopathy and COPD/asthma as comorbidities and a lower level of consciousness as a symptom. Cough and dyspnea were the most frequent symptoms in both groups (Table 1).

Based on clinical laboratory data collected within the first 24 hours, it was found that patients with COVID-19 had a lower mean partial pressure of oxygen/fraction of inspired oxygen (PaO_2_/FiO_2_) ratio than those without COVID-19 (212.7 *versus* 264.1; p < 0.001). COVID-19 patients had higher mean blood glucose, C-reactive protein, and lactic dehydrogenase levels than non-COVID-19 patients. Otherwise, higher white blood cell count, mean base excess, and median total bilirubin were observed in the non-COVID-19 group (Table 1).

When examining the data collected during ICU stay, we found that the COVID-19 group experienced a greater occurrence of hyperglycemia, acute renal failure, nosocomial infection, acute respiratory distress syndrome (ARDS) and more severe ARDS. In contrast, the non-COVID-19 group had a higher number of cases with pleural effusion and episodes of congestive heart failure (Table 2).

**Table 2:** Comparison of outcomes among patients admitted to the intensive care unit with acute respiratory failure due to COVID-19 and other causes

Outcomes	COVID-19 (n = 822)	Non-COVID-19 (n = 179)	p value
Complications			
Pleural effusion	53 (6.4)	27 (15.1)	< 0.001^*^
Convulsive crisis	6 (0.7)	2 (1.1)	0.639^*^
Stroke	8 (1.0)	4 (2.2)	0.244^*^
Congestive heart failure	8 (1.0)	15 (8.4)	< 0.001^*^
Endocarditis, myocarditis or pericarditis	3 (0.4)	0 (0)	1^*^
Arrhythmia	61 (7.4)	8 (4.5)	0.193^*^
Nosocomial infection	157 (19.1)	21 (11.7)	0.018^*^
Coagulopathy	377 (45.9)	83 (46.4)	0.934^*^
Acute renal failure (AKI-KDIGO stage 1, 2 or 3)	570 (69.3)	108 (60.3)	0.022^*^
Upper gastrointestinal bleeding	11 (1.3)	0 (0)	0.229^*^
Hepatic dysfunction (total bilirubin >1.2mg/dL)	143 (17.4)	29 (16.2)	0.744^*^
Pneumothorax	19 (2.3)	4 (2.2)	1^*^
Hyperglycemia (blood glucose ≥ 180mg/dL)	524 (63.8)	78 (43.6)	< 0.001^*^
Hypoglycemia (blood glucose < 70mg/dL)	200 (24.4)	36 (20.1)	0.245^*^
ARDS	774 (94.2)	114 (63.7)	< 0.001^*^
ARDS level			
Mild (did not use mechanical ventilation or always had a PaO_2_/FiO_2_ > 200 on mechanical ventilation)	353 (45.6)	63 (55.3)	< 0.001†
Moderate (used mechanical ventilation and the lowest PaO_2_/FiO_2_ was between 200 and 100)	152 (19.6)	35 (30.7)	
Severe (used mechanical ventilation and had one-time PaO_2_/FiO_2_ < 101)	269 (34.7)	16 (14.5)	
Inpatient treatments			
Oxygen by nasal catheter or mask	657 (80.0)	139 (77.7)	0.475^*^
Spontaneous pronation with oxygen support	100 (16.3)	6 (3.8)	< 0.001^*^
Noninvasive ventilation	36 (4.4)	12 (6.7)	0.181^*^
Mechanical ventilation	441 (53.6)	76 (42.5)	0.008^*^
Mechanical ventilation days^a^	9 (5 - 16)	7 (3 - 11.5)	0.001^*^
Pronation on mechanical ventilation	203 (28.4)	8 (4.6)	< 0.001^*^
Performed tracheostomy	63 (7.7)	12 (6.7)	0.755^*^
ECMO	2 (0.2)	0 (0)	1^*^
Performed dialysis	112 (13.6)	7 (3.9)	< 0.001^*^
Antiviral	402 (48.9)	123 (68.7)	< 0.001^*^
Antibiotic	755 (91.8)	169 (94.4)	0.281^*^
Corticoid	586 (71.3)	70 (39.1)	< 0.001^*^
Antifungal	188 (22.9)	16 (8.3)	< 0.001^*^
ICU outcome			
Days of ICU stay	6 (3 - 12)	4 (2 - 9)	< 0.001‡
Mortality	359 (43.7)	49 (27.4)	< 0.001^*^
Final diagnosis			< 0.001†
Not complicated with COVID-19	7 (0.9)	0 (0.0)	
COVID-19 pneumonia without ARDS	42 (5.1)	0 (0.0)	
COVID-19 pneumonia with ARDS	773 (94.0)	0 (0.0)	
Nonspecific pneumonia	0 (0.0)	64 (35.8)	
Specific pneumonia	0 (0.0)	21 (11.7)	
Others	0 (0.0)	94 (52.5)	
Clinical status on the 9-point ordinal scale			< 0.001†
0, 1 or 2 - Not hospitalized	21 (2.6)	15 (8.4)	
3 - Hospitalized, no oxygen therapy	195 (23.7)	53 (29.6)	
4 - Hospitalized, oxygen by mask or nasal prongs	247 (30.0)	61 (34.1)	
5 - Hospitalized, noninvasive ventilation or high-flow oxygen	0 (0.0)	0 (0.0)	
6 - Hospitalized, intubated and on mechanical ventilation	0 (0.0)	1 (0.6)	
7 - Hospitalized, on mechanical ventilation and additional organ support (renal replacement therapy, vasoactive drugs or ECMO)	0 (0.0)	0 (0.0)	
8 - Death	359 (43.7)	49 (27.4)	

ARDS - acute respiratory distress syndrome; PaO_2_ - partial pressure of oxygen; FiO_2_ - fraction of inspired oxygen; ECMO - extracorporeal membrane oxygenation; ICU - intensive care unit.

a Results considering only the number of participants who used mechanical ventilation.

* Fisher's exact test, p < 0.05 indicates statistical significance; † chi-square test, p < 0.05 indicates statistical significance; ‡ Non-parametric Mann-Whitney U test, p < 0.05 indicates statistical significance. Results expressed as n (%) or medians (first quartile - third quartile).

Patients with COVID-19 received significantly more pronation on mechanical and spontaneous ventilation, antiviral, antifungal, corticosteroid, and dialysis treatment, had a longer ICU stay (median 7 *versus* 4; p < 0.001), and had a higher mortality rate (43.7% *versus* 27.4%; p < 0.001) when compared to the non-COVID-19 group (Table 2).

**Figure 2:**
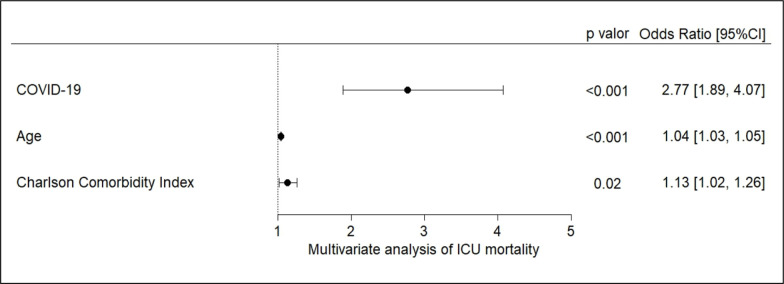
COVID-19 assessment as a predictor of intensive care unit mortality, regardless of age and Charlson Comorbidity Index score.

Among patients admitted to the ICU with acute respiratory failure secondary to suspected respiratory infection, patients with a confirmed COVID-19 diagnosis were 2.77 times more likely to die during their ICU stay than those without this diagnosis (95%CI, 1.89 to 4.07; p < 0.001), even when adjusted for age and Charlson Comorbidity Index score in a multivariate analysis. Greater age and Charlson Comorbidity Index score were also associated with an increased chance of ICU mortality (Figure 2).

Odds ratio and 95% confidence interval of the multivariate binary logistic regression model for intensive care unit mortality. p value: significance by Wald test. 95%CI - 95% confidence interval.

## DISCUSSION

In 2020, several patients were admitted to the ICU with symptoms of acute respiratory failure. Identifying which patients with acute respiratory failure had COVID-19 and/or were more likely to die on admission and providing the best care posed a challenge for clinicians. Here, we described a concomitant cohort of patients with acute respiratory failure with and without COVID-19 admitted to the ICU during the first wave of the pandemic in Brazil.^(14)^ In addition, we detailed the demographic, clinical, and laboratory characteristics, treatments, complications and outcome characteristics associated with these groups.

Throughout our analysis, COVID-19 alone significantly increased the mortality risk of patients. Our results also demonstrated a different profile of acute respiratory failure patients. For instance, the clinically relevant differences at baseline in COVID-19 patients were a higher body mass index, obesity, and test results showing higher blood glucose, C-reactive protein, and lactic dehydrogenase levels. Moreover, these patients were more likely to develop renal failure, hyperglycemia, ARDS, and a more severe level of ARDS. They used mechanical ventilation with the prone maneuver more frequently, had dialysis more frequently and had longer lengths of hospitalization.

On the other hand, the non-COVID-19 patients were older and had more comorbidities, such as cardiopathy and COPD/asthma. In the first 24 hours, higher values for leukocyte count, PaO_2_ and PaO_2_/FiO_2_ were observed. The outcome parameters observed were mostly moderate levels of ARDS, pleural effusion and congestive heart failure.

The baseline characteristics of the population studied were similar to those reported in other COVID-19 articles. Patients included in this cohort had a mean age similar to studies from Brazil and internationally.^(15-20)^ The most frequent comorbidities reported here are also compatible with the literature,^(15,18,19,21)^ including systemic arterial hypertension, diabetes and obesity. Importantly, non-COVID-19 patients presented more cardiopathy and COPD/asthma than COVID-19 patients. The APACHE II and SOFA scores reported in the literature thus far are very varied.^(17,22,23)^

Our sample of COVID-19 patients had less extrapulmonary organ dysfunction and more respiratory dysfunction than non-COVID-19 patients. The COVID-19 group had a lower mean PaO_2_/FiO_2_ ratio than the non-COVID-19 group. Similar findings by Kurtz et al.^(23)^ showed that approximately 50% of COVID-19 patients had a PaO_2_/FiO_2_ ratio lower than 199. The SOFA scores on admission were lower than that in previous reports,^(17,18)^ which could be related to the patients’ demographic characteristics in this study. As expected, due to the extensive inflammatory component of this disease, C-reactive protein levels were higher in COVID-19 patients and could be an important biomarker at admission.^(24)^

In this cohort, the COVID-19 group had a 30.85% greater proportion of people with ARDS and two times more severe ARDS levels than the non-COVID-19 group. Other complications observed in the COVID-19 group were acute renal failure, hyperglycemia and nosocomial infection, compatible with the COVID-19 pathophysiology and clinical findings.^(5,25-27)^ Regarding pulmonary parameters, there is substantial variation in the use of mechanical ventilation in COVID-19 patients in the literature,^(17-20,28-30)^ which could reflect differences in clinical practice and patient profiles. However, mechanical ventilation is more common in COVID-19 patients than in non-COVID-19 patients with acute respiratory failure. The days of ICU stay were similar to those in other studies in the COVID-19 group.^(15,19,30)^

Reported mortality rates are highly variable in studies on COVID-19, ranging from 10.8 to 55%.^(15-17,23)^ Our results are similar to those in studies in Brazil^(15,16)^ but still higher than the results of a meta-analysis performed worldwide (33%). Patients’ characteristics at admission could explain these discrepancies, such as APACHE II and SOFA scores, as mentioned before. A Brazilian study^(15)^ obtained similar data from ICU COVID-19 patients regarding mortality, the need for mechanical ventilation, symptoms and ICU stay, demonstrating a similar profile of patients in this region. Additionally, mortality among COVID-19 patients was 16% higher than that among non-COVID-19 patients.

The main strength of this study is the concomitant comparison between COVID-19 and non-COVID-19 patients in a large sample of ICU patients. Moreover, we performed a more complete analysis of these patients with information related to patient laboratory results, treatments, complications, and outcome characteristics that can be useful to the growing literature on COVID-19.

However, there are also some limitations to this study. First, data collection was restricted to the tests that were ordered in the ICU and documented in the electronic medical records. As a result, 17.8% of eligible patients were excluded due to insufficient medical records, which could have introduced some selection bias into this study. The electronic records were developed promptly and prospectively; however, data were gathered only from Curitiba, Brazil, at the start of the COVID-19 outbreak. During that time, actions and interventions were still being studied and learned, so the sample was not representative of the entire country during the pandemic. It is possible that the outcome may have been influenced by changes in clinical management and other interventions, such as the use of steroids, anticoagulation, and others over time. Finally, there was an important imbalance in the number of patients in each group, which may have influenced the results; however, this imbalance reflected the pandemic context.

Finally, we contribute to the description of the phenotypes of patients treated in the ICU for acute respiratory failure due to COVID-19 and other causes in the Brazilian context, and we believe that the results of our cohort add specific information to the currently available literature detailing the differences and similarities of these groups concurrently in relation to a wide range of parameters relevant to clinical decision-making for critically ill patients.

## CONCLUSION

Patients with acute respiratory failure secondary to COVID-19 had a clearly different phenotype than non-COVID-19 patients and had a higher risk of dying in the intensive care unit than those without COVID-19, even when adjusted for age and comorbidities. Knowing the key differences between patients with COVID-19 and those without COVID-19 can contribute to informing multidisciplinary teams about the management of new patients and help to delineate more accurate screening and appropriate and timely treatment for these patients.
